# Proteomic Response of *Pseudomonas putida* KT2440 to Dual Carbon-Phosphorus Limitation during mcl-PHAs Synthesis

**DOI:** 10.3390/biom9120796

**Published:** 2019-11-28

**Authors:** Justyna Możejko-Ciesielska, Luísa S. Serafim

**Affiliations:** 1 Department of Microbiology and Mycology, Faculty of Biology and Biotechnology, University of Warmia and Mazury in Olsztyn, Oczapowskiego 1A, 10719 Olsztyn, Poland; 2 Chemistry Department, CICECO-Aveiro Institute of Materials, University of Aveiro, Campus Universitário de Santiago, 3810-193 Aveiro, Portugal; luisa.serafim@ua.pt

**Keywords:** mcl-polyhydroxyalkanoates, phosphorus limitation, proteome, *Pseudomonas putida* KT2440

## Abstract

*Pseudomonas putida* KT2440, one of the best characterized pseudomonads, is a metabolically versatile producer of medium-chain-length polyhydroxyalkanoates (mcl-PHAs) that serves as a model bacterium for molecular studies. The synthesis of mcl-PHAs is of great interest due to their commercial potential. Carbon and phosphorus are the essential nutrients for growth and their limitation can trigger mcl-PHAs’ production in microorganisms. However, the specific molecular mechanisms that drive this synthesis in *Pseudomonas* species under unfavorable growth conditions remain poorly understood. Therefore, the proteomic responses of *Pseudomonas putida* KT2440 to the limited carbon and phosphorus levels in the different growth phases during mcl-PHAs synthesis were investigated. The data indicated that biopolymers’ production was associated with the cell growth of *P. putida* KT2440 under carbon- and phosphorus-limiting conditions. The protein expression pattern changed during mcl-PHAs synthesis and accumulation, and during the different physiological states of the microorganism. The data suggested that the majority of metabolic activities ceased under carbon and phosphorus limitation. The abundance of polyhydroxyalkanoate granule-associated protein (PhaF) involved in PHA synthesis increased significantly at 24 and 48 h of the cultivations. The activation of proteins belonging to the phosphate regulon was also detected. Moreover, these results indicated changes in the protein profiles related to amino acids metabolism, replication, transcription, translation, stress response mechanisms, transport or signal transduction. The presented data allowed the investigation of time-course proteome alterations in response to carbon and phosphorus limitation, and PHAs synthesis. This study provided information about proteins that can be potential targets in improving the efficiency of mcl-PHAs synthesis.

## 1. Introduction

Polyhydroxyalkanoates (PHAs) have attracted much attention in recent years due to their potential to replace petroleum-based polymers. Many bacteria were already reported to be capable of synthesizing PHAs. PHAs are mainly accumulated intracellularly as a storage material for carbon and energy when essential nutrients are in limiting concentrations. The suitability of a bacterium for PHA production depends on many different factors, such as the safety of the organism, achievable cell densities, and the amount, composition, extractability, and properties of accumulated PHAs. PHA monomers produced by microorganisms are classified into three groups: Short chain length (scl-PHA), which consists of monomers containing 4 to 5 carbon atoms, medium chain length (mcl-PHA) containing 6 to 14 carbon atoms, and long chain length with more than 15 carbon atoms [1]. In particular, mcl-PHAs are mainly synthesized and accumulated by *Pseudomonas* species and are a subject of interest because of their improved properties when compared with scl-PHAs. Due to these properties, mcl-PHAs are promising materials in various fields, especially in biomedical applications, such as tissue engineering and drug delivery [2].

*Pseudomonas putida* KT2440 is considered a model microorganism for molecular studies since the publication of its complete genome [3]. Due to the classification as host-vector system safety level 1 (HV1) certified, broad metabolic versatility, and genetic plasticity, this strain is widely used in improving the understanding of the fundamental biology of *Pseudomonas* species [4]. Moreover, the availability of the *P. putida* KT2440 genome, together with advances in molecular techniques and computational tools, allows the provision of knowledge about metabolic and regulatory networks that drive the synthesis of mcl-PHAs.

Although the technological aspects of PHAs production, including bacterial production, extraction, purification, and physicochemical characterization, were extensively studied during the past few decades, knowledge on the metabolic networks responsible for mcl-PHAs synthesis at the molecular level is relatively limited. Six proteins directly involved in the biosynthesis and degradation of mcl-PHAs have already been characterized: Two polymerases, PhaC1 and PhaC2; a depolymerase, PhaZ; two phasins, PhaI and PhaF; and two regulatory proteins, PhaD and PhaG. PhaC1, PhaC2, PhaZ, and PhaD are encoded by genes transcribed in the same direction, whereas PhaF and PhaI are transcribed in the opposite way [5]. The expression of *pha* genes is controlled by global transcriptional regulators, such as Crc, RpoS, PsrA, and GacS/GacA systems. The regulatory network that could drive the PHA cycle in pseudomonads was reviewed in detail by Prieto et al. [6]. PhaG encodes transacylase, which is not co-localized with the PHA biosynthesis gene cluster [7]. Możejko-Ciesielska et al. [8] suggested that the *phaG* gene could be associated with the mcl-PHAs synthesis process not only on non-related carbon sources but also on related ones.

The regulatory mechanisms that drive PHA synthesis in *Pseudomonas* species were previously studied at the proteomic level. However, the molecular basis behind microbial mcl-PHAs only under nitrogen-, carbon-, and carbon-nitrogen limitation was already explored [9,10,11,12]. Because phosphorus is an essential macroelement for all microorganisms, its limitation can have a major effect on cellular metabolism and physiology. Phosphorus is involved in nucleic acid metabolism, cell membrane synthesis, and signal transduction [13]. Bacteria have the ability to control the mechanisms engaged in sensing phosphate availability as well as phosphate uptake and utilization. These networks are usually related with central metabolic routes because of the importance of phosphate in cellular physiology. Eberl et al. [14] demonstrated that *Pseudomonas putida* KT2442 cells retained a rod shape under phosphate-deprived conditions whereas carbon-starved cells formed spherical cells as a result of reductive cell divisions. Furthermore, protein synthesis and ribosome concentration were gradually reduced, and ATP levels dropped to very low values after the onset of starvation; later, however, there was a return to near-normal ATP concentrations. Moreover, there is evidence that the phosphate deprivation regulon is involved in influencing virulence traits in bacteria [15]. It is known that bacteria are able to synthesize high levels of PHA under phosphorus limitation [16,17,18] and a dual nutrient-limiting regime [10]. However, it is not known how *Pseudomonas* species respond to dual carbon-phosphorus limitation during mcl-PHAs synthesis and accumulation.

The aim of this study was to gain insights into the proteomic response of *Pseudomonas putida* KT2440 to dual carbon-phosphorus limitation. Two-dimensional electrophoretic protein separation (2DE) and mass spectrometry analysis (MALDI-TOF/TOF) enabled deeper and more accurate analysis of the proteome at the different *P. putida* KT2440 growth stages during medium-chain-length polyhydroxyalkanoates synthesis.

## 2. Results and Discussion

### 2.1. The Influence of Phosphorus Availability on Growth, and mcl-PHAs Synthesis and Composition

**Figure 1:**
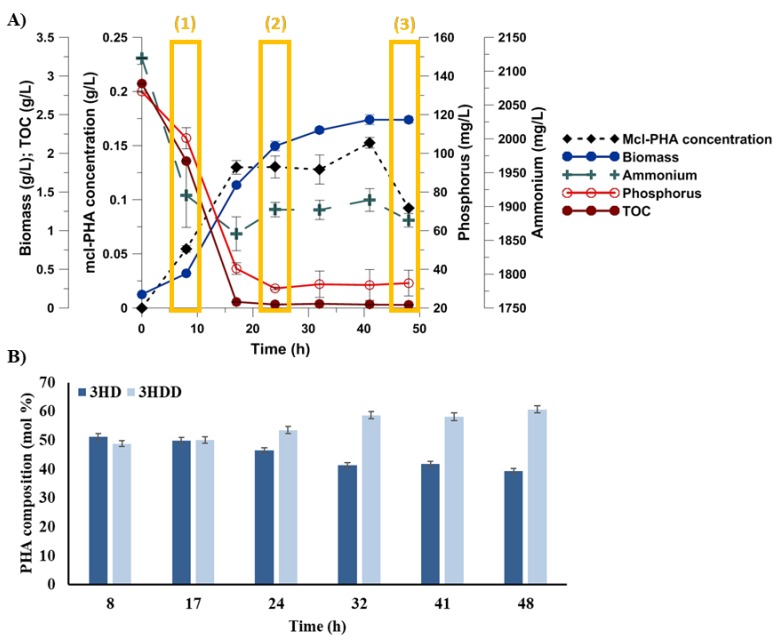
Parameters measured during the cultivation of *Pseudomonas putida* KT2440 towards medium-chain-length polyhydroxyalkanoates under dual carbon-phosphate limitation. The mean was taken from three biologically independent replicates. (**A**) Mcl-PHAs content, biomass, total organic carbon (TOC), phosphorus, and ammonium concentration during the fermentations. (1), (2) and (3) indicate samples collected at 8, 24, and 48 h of cultivation, respectively. (**B**) Monomeric composition of synthesized mcl-PHAs in bioreactor experiments. 3HD, 3-hydroxydecanoic acid; 3HDD, 3-hydrododecanoic acid.

To evaluate the cellular response to carbon and phosphorus limitation during mcl-PHAs biosynthesis, *Pseudomonas putida* KT2440 cultivation was conducted for 48 h. Bacterial growth, carbon, phosphorus, and ammonium utilization, and mcl-PHAs synthesis were monitored during cultivations (Figure 1A). The ammonium concentration remained above 1.8 g/L through the whole cultivation. The phosphorus was consumed during the bacterial growth, reaching about 30 ± 6.7 mg/L at the end of the fermentation. Also, the carbon in the culture broth was consumed during the growth and was almost depleted after 17 h. The biomass concentration gradually increased up to 41 h, and then slowed down, reaching 2.4 ± 0.04 g/L at 48 h. The biopolymers were synthesized and accumulated within the cells during the exponential growth phase. It was previously proved that during this phase, *P. putida* cells increase their size, which could be attributed to the accumulation of biopolymers intracellularly [14]. From 41 h of cultivation, the mcl-PHAs concentration decreased up to the late stationary phase, reaching only 0.09 ± 0.001 g/L at the end of the culture. The lowest levels of carbon and phosphorus reached between 17 and 41 h of the cultivation hindered mcl-PHAs biosynthesis. Moreover, biopolymer production was associated with the cell growth of *P. putida* KT2440 under the applied unfavorable environmental conditions. Previously, a positive correlation between the biomass and biopolymer concentration in a cultivation of the same bacterial strain was also observed under nitrogen-limitation conditions [19].

*Pseudomonas putida* KT2440 was also cultivated in a bioreactor under non-limiting conditions. Our results revealed that during optimal growth conditions, *P. putida* was not able to synthesize PHAs (Table S1). The obtained data proved that the carbon and phosphorus concentration in the limiting medium was low enough to support mcl-PHAs synthesis and accumulation.

The gas chromatography analysis revealed that the synthesized mcl-PHAs had the same repeat-unit compositions during the whole fermentation process (Figure 1B). The composition of mcl-PHAs produced by the *P. putida* KT2440 was found to contain only 3-hydroxydecanoic acid (3HD) and 3-hydroxydodecanoic acid (3HDD). The major repeat units were similar to those produced by *P. putida* KT2440 cultivated on citrate towards biopolymer synthesis [20]. At 8 h, the proportion of the two monomers was similar, 51.2% ± 1.09% for 3HD and 48.8% ± 1.06% for 3HDD. Along the fermentation time, the amount of 3HDD increased to 60.7% ± 1.18%, meaning that 3HD was preferentially consumed, when PHA started to be used by bacterial cells.

### 2.2. Overview of Proteomic Analysis

**Figure 2:**
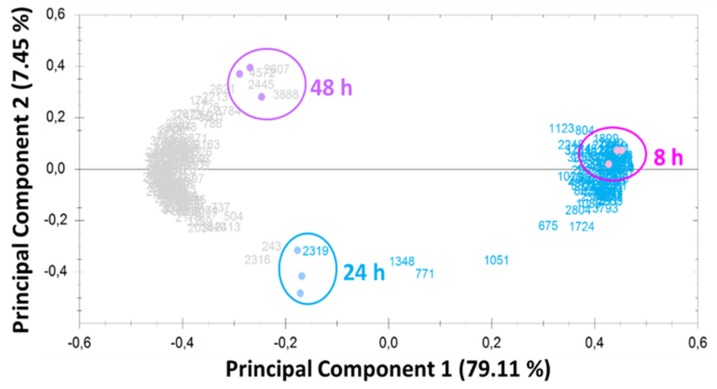
Principal component analysis was calculated by Same Spots software. Dot plot of all spots included in the analysis shows that in all three experimental sets (at 8, 24, and 48 h), replicate samples group are close together.

To understand *P. putida* KT2440 cell growth and mcl-PHAs synthesis in response to dual carbon and phosphorus limitation, the proteome profiles were examined at various growth phases of the bacterial cells and at different biopolymers’ accumulation stages. These sampling points corresponded to the early exponential phase and mcl-PHA production (8 h of cultivation), to the late exponential phase (24 h of cultivation) and late stationary growth phase (48 h of cultivation). It was expected that not only mcl-PHAs synthesis and accumulation but also the different physiological states and nutrient limitation during fermentation would most likely affect the protein expression pattern. Principal component analysis (PCA, Figure 2) of all the differentially expressed spots included (Table S2) shows that replicate samples at each time point are highly similar in all cases and that protein expression patterns at 8, 24, and 48 h are easily distinguished from each other.

**Figure 3:**
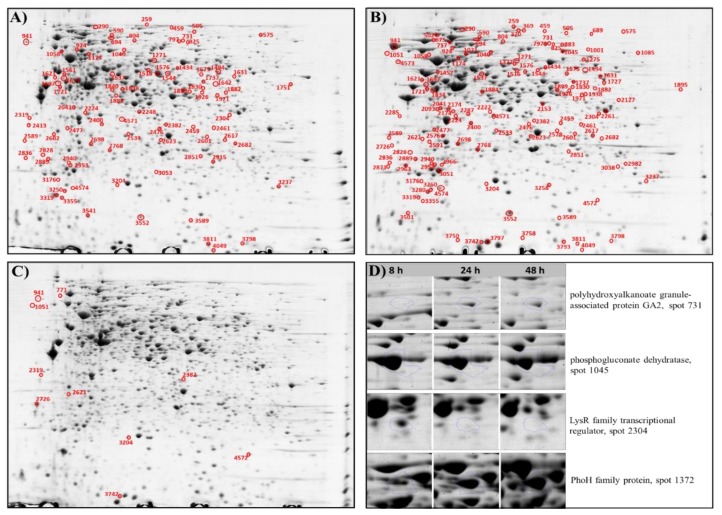
Representative 2D electrophoresis gel images of *Pseudomonas putida* KT2440 at three time points. The successfully identified differentially significantly expressed proteins are labeled on the images in the red color. (**A**) 8 versus 24; h (**B**) 8 versus 48 h; (**C**) 24 versus 48 h (**D**) Examples of normalized 2DE spots.

Figure 3 shows representational gel maps of the proteins isolated from *P. putida* KT2440 cells at the analyzed time points of the fermentation. Based on the comparison and analysis of the gel images, a total of 135 significantly differentially expressed proteins were successfully identified using MALDI-TOF/TOF analysis (Table S2).

**Figure 4:**
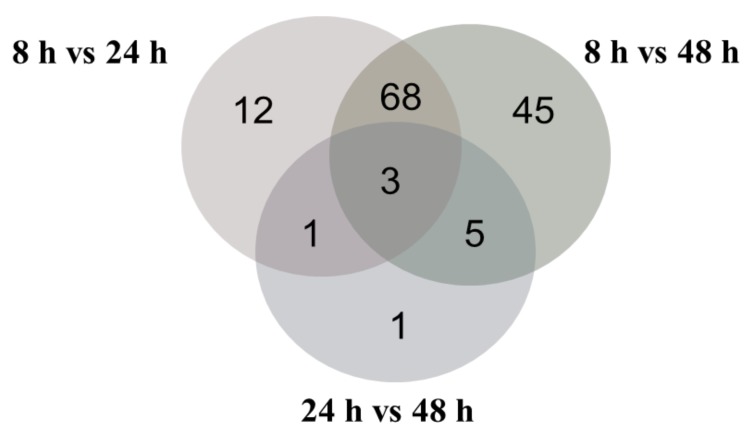
Number of common and specific significantly differentially expressed proteins during mcl-PHAs synthesis under phosphate limitation.

The results showed that 84 and 121 protein spots had a significantly altered expression at 24 and 48 h (relative to 8 h of growth), respectively. The proteomic analysis showed that 45 and 68 proteins were significantly downregulated, and 39 and 53 proteins were significantly upregulated at 24 and 48 h, respectively. Only 10 proteins were differentially regulated at 24 h compared to 48 h of the bacterial growth, 4 proteins were downregulated and 6 of them were upregulated. In total, 69 proteins that were differentially regulated at 24 h as compared to the beginning of the culture also had altered expression at 48 h. The analysis made between all analyzed variants revealed that only three common proteins with altered expression were observed (Figure 4).

### 2.3. Proteome Changes during mcl-PHAs Synthesis and Different Growth Phases

#### 2.3.1. Carbon and Energy Metabolism

To gain more insights on mcl-PHAs accumulation under dual carbon-phosphorus limitation, the abundance changes of proteins related to carbon metabolism were analyzed. To support the growth of bacterial cells, sodium gluconate was used as the only carbon source. External gluconate transported by GnuK can be metabolized to pyruvate and glyceraldehyde 3-phosphate via the Entner–Doudoroff (ED) route or can be converted to 6-phosphogluconate and then decarboxylated to pentose-phosphate. The results confirmed that Edd protein (spot 1045; *p*-value = 9.894 × 10^−5^) was present at the highest level in the beginning of the process. Then, its repression was observed at 48 h when mcl-PHAs were not synthesized, indicating that this protein could affect the intracellular biopolymers’ accumulation. These data are consistent with previous RNA-seq analysis, confirming that genes related to the ED pathway could be potential targets in improving mcl-PHAs synthesis [8].

Furthermore, one of the key enzymes involved in glycolytic pathway, enolase (spot 1697; *p*-value = 0.018), was significantly induced in the late exponential and stationary phase. However, at 48 h of cultivation, the expression of glyceraldehyde-3-phosphate dehydrogenase (spot 2261; *p*-value = 9.983 × 10^−5^) was repressed by 2.1-fold, suggesting a decreased production of pyruvate, and as a consequence less acetyl-CoA, could be provided for the bacterial cells. In this study, the abundance of isocitrate dehydrogenase (spot 694; *p*-value = 4.449 × 10^−4^) involved in the tricarboxylic acid cycle (TCA) also decreased after 8 h of cultivation. Moreover, acetyl-CoA carboxylase biotin carboxyl carrier protein subunit (AccB; spot 4574; *p*-value = 5.352 × 10^−5^) presented a 3.9-fold and 4.0-fold repression at 24 and 48 h, respectively. This is the first enzyme of fatty acid biosynthesis, leading the initial step from acetyl-CoA to fatty acid de novo synthesis via malonyl-CoA while growing on non-related carbon sources, like gluconate. This enzyme was considered as being involved in PHAs synthesis [10]. In the present study, the abundance of AccB decreased when bacterial growth reached the late exponential phase and mcl-PHAs synthesis slowed down in the bacterial cells due to limiting conditions.

*Pseudomonas putida* KT2440 synthesized and accumulated mcl-PHAs up to 41 h of its cultivation. Then, the bacteria stopped the biopolymers’ synthesis, and carbon and phosphorus in cells were consumed to their lowest point. Thus, the proteins involved in the energy generation process began to be downregulated, such as FOF1 ATP synthase subunit beta (spot 1457; *p*-value = 0.012), glutamate dehydrogenase (spot 1737; *p*-value = 1.176 × 10^−5^), glutamine synthetase (spot 1174; *p*-value = 2.196 × 10^−4^), NADH dehydrogenase subunit G (spot 370; *p*-value = 0.048), and nitrate reductase (spot 2533; *p*-value = 5.195 × 10^−4^).

#### 2.3.2. PHA Biosynthesis 

PHA biosynthetic protein (polyhydroxyalkanoate granule-associated protein GA2, PhaF, spot 731; *p*-value = 3.157 × 10^−5^) was detected as significantly differentially expressed. This protein was reported to coat PHA hydrophobic inclusions, playing an important role in PHA biosynthesis and PHA granule biogenesis [21]. Furthermore, PhaF was recognized as a multifaceted protein, being essential in the granule localization and ensuring an equal distribution between daughter cells during cell division by a simultaneous attachment to the PHA polymer and to nucleoid DNA [22]. In addition, as a phasin, it could work as a protector against damage of cell components due to interactions with the polymer [23]. In this study, the abundance of PhaF protein increased at 24 and 48 h relative to 8 h of *P. putida* KT2440 culture; thus, when the mcl-PHAs content in bacterial cells was higher. Galan et al. [24] suggested that PhaF could contribute to the optimization of the intracellular PHA content. Moreover, this protein seems to be involved in the transcriptional regulatory system as a negative regulator of the *pha* cluster in *P. putida* GPo1 [25]. In *P. putida* KT2440, the deletion of the *phaF* gene resulted in an increased *pha* cluster transcription rate when cells were cultured in batch fermentation, while the PHA content remained unaltered [25]. The same authors revealed that during continuous culture, the disruption of PhaF phasin affected considerably the PHA content of the cells. In our study, other PHA-related proteins, like PhaC1, PhaZ, PhaC2, PhaD, and PhaI, were not identified as being differentially abundant. This could suggest that their concentration was too low to be detected using 2DE electrophoresis analysis.

#### 2.3.3. Amino Acid Metabolism and Biosynthesis

Amino acid catabolism is especially necessary under nutritional stress situations. Under these conditions, amino acids are used as alternative substrates to produce energy under unfavorable growth conditions. An increased abundance of 3-hydroxyisobutyrate dehydrogenase (spot 2476; *p*-value = 2.207 × 10^−5^), leucine dehydrogenase (spot 1971; *p*-value = 2.875 × 10^−4^), cysteine synthase (spot 2222 and 459; *p*-value = 9.648 × 10^−4^ and 6.696 × 10^−4^, respectively), and phosphoribosyl-AMP cyclohydrolase (spot 2889; *p*-value 7.960 × 10^−6^) was observed at 24 and 48 h of the bioprocess. 3-hydroxyisobutyrate dehydrogenase is essential for valine metabolism, and catalyzes a reversible oxidation of L-3-hydroxyisobutyrate to methylmalonate semialdehyde [26]. Leucine and valine concentrations increase under various stress conditions and their complete oxidation allows the generation of high amounts of ATP [27,28]. Furthermore, the protein expression profile showed that other differentially expressed proteins involved in amino acid metabolism were downregulated. The abundance of 3-isopropylmalate dehydrogenase (spot 1071; *p*-value = 2.920 × 10^−4^), aminotransferase (spot 1899; *p*-value = 5.384 × 10^−4^), diaminopimelate epimerase (spot 2623; *p*-value = 2.040 × 10^−5^), N-acetyl-gamma-glutamyl-phosphate reductase (spot 2461; *p*-value = 0,009), hydantoinase B/oxoprolinase (spot 1040; *p*-value = 0.001), and oxoprolinase (spot 797; *p*-value = 9.876 × 10^−6^) participating in the biosynthesis of leucine, lysine, arginine, and proline decreased from 2.1-fold to 4.5-fold at 24 and 48 h relative to 8 h.

#### 2.3.4. Stress Response

Proteins that are generally regarded as stress responsive were also differentially regulated under nutrient limitation during mcl-PHAs synthesis. The obtained data confirmed that *P. putida* KT2440 responds to phosphorus stress conditions by activating PhoH family protein (spot 1372; *p*-value = 2.226 × 10^−4^) belonging to the phosphate regulon. Furthermore, the abundance of chaperonin GroEL (spot 1058; *p*-value = 0.006), universal stress protein (spot 3811), and quinoprotein ethanol dehydrogenase (spot 1001; *p*-value = 0.010) increased significantly during the time-course of biopolymer synthesis. GroEL, as the chaperonin family of molecular chaperones, is involved in the proper folding of many proteins [29]. Therefore, its increased abundance during *P. putida* KT2440 growth was important for aggregated proteins to renature after exposure to unfavorable conditions. A similar situation was observed during cellular proteome alterations of *Pseudomonas putida* to naphthalene-induced stress [30]. Furthermore, the upregulation of quinoprotein ethanol dehydrogenase protein could be related to the protection against the abnormal formation of reactive oxygen species (ROS). These data are in agreement with the results obtained by Kurbatov et al. [31], who observed a significant abundance of the above-mentioned protein induced during stressful growth conditions of *P. putida* KT2440 grown on phenol. In contrast, chaperone protein HscA (spot 804; *p*-value = 0.010), chaperone protein DnaK (spot 675; *p*-value = 0.028), heat shock protein Hsp20 (spot 3793; *p*-value = 3.776 × 10^−5^), anti-oxidant AhpCTSA family protein (spot 3051; *p*-value = 3.906 × 10^−5^), and peptidyl-propyl cis-trans isomerase, FKBP-type (spot 2963; *p*-value = 7.963 × 10^−4^) were downregulated in the late exponential and stationary growth phases. The highest expression of these proteins was observed at the beginning of the process when the mcl-PHAs started to be produced. DnaK is involved both in co- and post-translational folding processes, whereas HscA seems to be specialized for the assembly of iron-sulfur cluster proteins [32,33]. Genetic studies indicated that the deletion of the *dnaK* and *hscA* genes revealed that they are not absolutely essential for bacterial survival. *Escherichia coli* double mutants lacking these genes were still viable and any defects in protein folding were observed during its growth [34]. In the present study, the abundance of polynucleotide phopshorylase/ polyadenylase protein (PNPase, spot 717; *p*-value = 0.002) decreased but only at 48 h compared to 8 h. This protein is related to the mRNA degradation system in bacteria [35]. Yehudai-Resheff et al. [36] assumed that a high concentration of phosphorus in bacteria enhanced PNPase degradation activity. It could be suggested that this protein was repressed due to the limited phosphorus level during the process.

Moreover, the proteins related to signal transduction, the LytTR family two component transcriptional regulator (spot 2578; *p*-value = 0.002), sensor histidine kinase (spot 575; *p*-value = 5.189 × 10^−6^), and LuxR family two component transcriptional regulator (spot 2966; *p*-value = 0.006) were upregulated at the late exponential phase when compared to the early exponential one. The LytTR regulatory system was demonstrated to respond to unusual growth conditions and environmental stress [37]. In the present study, its expression could be triggered by the dual carbon and phosphorus limitation.

#### 2.3.5. Replication, Transcription, and Translation

The analysis of the identified proteins showed a clear enrichment of proteins belonging to transcription and translation. One of the ways in which bacteria respond to environmental stress is through posttranslational modifications of translation factors. Elongation factor thermo unstable (Ef-Tu) plays a crucial role in protein biosynthesis, as it is methylated and phosphorylated in response to nutrient starvation upon entering the stationary phase [38]. A significant upregulation of elongation factor Tu was observed (spot 505; *p*-value = 2.620 × 10^−6^) in the stationary phase when compared to the beginning of the bioprocess. Furthermore, the expression of elongation factor Tu-A (spot 1561; *p*-value = 0.003) increased by 6.7-fold at 24 h relative to 8 h and was downregulated by 2.3-fold in the late stationary stage. In addition, elongation factor 4 (spot 4572; *p*-value = 4.769 × 10^−4^), thought to be a protective protein from stress, showed upregulation of a weak but significant nature in the stationary phase. The experimental results suggest that transcription elongation factor GreA (spot 3280; *p*-value = 5.932 × 10^−4^), and NusA (spot 771; *p*-value = 0.020) were significantly repressed at 24 and 48 h, respectively. GreA is involved not only in the transcription regulation but also in the protection of cellular proteins against aggregation [39]. Additionally, the abundance of a key coordinator of microbial gene expression, LysR family transcriptional regulator (spot 2304; *p*-value = 3.556 × 10^−5^), decreased significantly by 6.2- and 6.4-fold at 24 and 48 h of the bioprocess, respectively. The above-mentioned data could suggest a general decrease in gene expression under dual carbon and phosphorus limitation that could also inhibited ribosome synthesis. During ribosome degradation, a large amount of ribosomal RNAs could be degraded [13].

Also, proteins as DNA gyrase subunit B (spot 369; *p*-value = 5.060 × 10^−5^) and single-strand DNA-binding protein (spot 3258; *p*-value = 1.354 × 10^−4^), essential for DNA replication and transcription, were significantly repressed at 48 h compared to the 8 h of the bioprocess under the applied stress conditions. There is a link between gyrase and cell division since decreasing gyrase levels can affect cell growth and cause chromosome relaxation and altered gene expression [40]. The experimental results showed a decreased abundance of DNA gyrase along with the repression of cell division protein FtsZ (spot 1834; *p*-value = 4.788 × 10^−4^) that influenced the low *P. putida* KT2440 growth level and as a consequence the low content of mcl-PHAs in bacterial cells. These observations were previously described by Guha et al. [41], who showed that gyrase depletion altered ftsZ expression in *Mycobacterium smegmatis*, leading to slower growth. Furthermore, at the same time point (48 h), the abundance of DNA-3-methyladenine glycosylase (spot 2591; *p*-value = 0.003) was increased. This protein participates in the initiation of the base excision repair process of damaged DNA [41].

#### 2.3.6. Transport

Aside from genes related to the stress response, a number of transporters were differentially regulated during mcl-PHAs synthesis under dual carbon- and phosphorus-limiting conditions. The transport of molecules across cellular membranes is essential for bacteria to maintain an off-equilibrium condition [42]. Furthermore, transporters play an important role not only in the uptake of nutrients but also in the maintenance of cell integrity, response to environmental stresses, or cell-to-cell communication [43]. Several transporters were detected in the present study and were upregulated in the late exponential and stationary phase compared to the early exponential phase of the *P. putida* KT2440 growth, namely, ABC transporters responsible for amino acid uptake (spots 1840, 2287, 2602; *p*-value = 0.011, 1.056 × 10^−4^, 1.013 × 10^−4^, respectively), dipeptide transport (spot 1275; *p*-value = 0.003), polyamine transport (spot 2127; *p*-value = 0.021), polysaccharide export protein (spot 3355; *p*-value = 3.863 × 10^−4^), and OmpF family protein (spot 2726, 2836, 2413, 2319, 2871; *p*-value = 0.003, 0.002, 0.029, 0.043, 0.013, respectively). The presence of these transporters shows that the physiological and biochemical processes during carbon metabolism and mcl-PHAs synthesis were increased. The abundance of the iron ABC transporter, periplasmic iron-binding protein (spot 2174; *p*-value = 0.023), decreased at 48 h when compared to 8 h of cultivation. Furthermore, translocation protein TolB (spot 1895; *p*-value = 0.001), being responsible for maintaining outer membrane integrity, was upregulated in the late stationary phase relative to the beginning of the bioprocess. Its highest expression is related to protective role of bacterial cells from stress-induced substances or degradation by digestive enzymes, while allowing the uptake of nutrients for their growth [44].

#### 2.3.7. Other Proteins 

Other proteins that did not fit in any of the previous classification were detected during the proteomic analysis. One of them was UDP-3-O-[3-hydroxymyristoyl] N-acetylglucosamine deacetylase (spot 2041; *p*-value = 2.917 × 10^−4^) that catalyzes the second step in the biosynthesis of lipid A that is required for bacterial growth, and inhibition of its biosynthesis is lethal to bacteria [45]. Its abundance increased along the analyzed time points. The upregulation of bacterioferritin (spot 3319; *p*-value = 0.009) was also observed in the stationary phase of the *P. putida* KT2440 growth. This protein is involved in iron binding and can serve to ease iron-related oxidative stress [46]. The proteomic analysis of *Pseudomonas putida* CA-3 revealed that a high expression level of bacterioferritin was induced under nitrogen-limited growth conditions on styrene during mcl-PHAs synthesis [9]. Furthermore, the proteomic results showed that the abundance of PhaZ family phenazine biosynthesis protein (spot 2621; *p*-value = 5.117 × 10^−6^) increased at 48 h relative to the beginning of the bioprocess. Recently, phosphate starvation during *P. aeruginosa* growth was proven to led to PhoB induction of the expression of phzA1/2 (phenazine biosynthesis). PhoB belongs to the Pho regulon that plays a regulatory circuit of phosphate homeostasis and is important in the adaptation to the stress response [47].

## 3. Material and Methods

### 3.1. Cultivation and Analytical Procedures

*Pseudomonas putida* KT2440 (ATCC^®^ 47054™) from long-term storage was firstly cultivated in nutrient-rich lysogeny broth (10g/L tryptone, 5 g/L yeast extract, 10 g/L NaCl) at 30°C at 220 rpm in a rotary shaker. Then, the bacterial cells were grown in a 7-L bioreactor (Biostat A, Sartorius, Germany) with a working volume of 5.0 L. The inoculation volume was 10%. The pH-value was controlled at 7 through the modulated addition of concentrated 1 N NaOH and 1 N HCl. The dissolved oxygen concentration was monitored with an O_2_ electrode (InPro 6800, Mettler Toledo GmbH, Switzerland) and controlled at least at 50% of saturation by adjusting the agitation rate from 300 to 800 rpm automatically and an airflow of 200 L/h. The phosphorus-limited mineral medium contained (per liter): 1.0 g Na_2_HPO_4_·12H_2_O, 0.2 g KH_2_PO_4_, 10 g (NH_4_)SO_4_, and 1 g MgSO_4_·7H_2_O; whereas the non-limiting medium consisted of (per liter): 3.5 g Na_2_HPO_4_·12H_2_O, 7.0 g KH_2_PO_4_, and 10 g (NH_4_)SO4. After being sterilized at 121 °C for 15 min., the media were supplemented with 10 g/L of sodium gluconate as a carbon source and 2.5 mL of trace element solution. The trace element solution contained per liter: 20 g FeCl_3_·6H_2_O, 10 g CaCl_2_·H_2_O, 0.03 g CuSO_4_·5H_2_O, 0.05 g MnCl_2_·4H_2_O, and 0.1 g ZnSO_4_·7H_2_O dissolved in 0.5 N HCl. Antifoam *A concentrate* (Sigma Aldrich, USA) was used to control foam formation. Total fermentation time was 48 h.

During the fermentation, the optical density of cells at 600 nm was measured to monitor the growth of the analyzed strain. Moreover, samples of 50 mL of the culture broth were periodically harvested for measurements of dry cell weight, mcl-PHAs, phosphorus, and ammonium concentration and for determination of the monomer composition. To determine the dry cell weight (DCW), 100 mL of culture broth were taken from the bioreactor, centrifuged at 11,200× *g* for 10 min, lyophilized, and weighted. The lyophilization process was performed in a Lyovac GT2 System (SRK Systemtechnik GmbH) for 24 h. The monomeric composition of the mcl-PHAs was determined by gas chromatography (GC Varian CP-3800) equipped with a capillary column Varian VF-5 ms with a film thickness of 0.25 mm (Varian, Lake Forest, USA). The concentration of methyl esters was determined using the lyophilized bacterial cells and following the methanolysis protocol described previously [48]. Ammonium, phosphorus, and total organic carbon (TOC) concentration were measured spectrophotometrically using the Hach Lange DR 2800 spectrophotometer (Hach Lange, Düsseldorf DE), and the LCK303, LCK350, and LCK380 kit according to the manufacturer’s instructions, respectively.

### 3.2. Protein Extraction and Two-Dimensional Gel Electrophoresis (2-DE)

For proteomic analysis, *Pseudomonas putida* KT2440 cells were taken from the bioreactor at 8, 24, and 48 h and were centrifuged for 5 min at 4 °C (10,000× *g*). After centrifugation, proteins were precipitated using the 2-D Clean-Up Kit (GE Healthcare, Uppsala, Sweden) according to the manufacturer’s protocol. The protein concentration was measured by the Coomassie (Bradford) Protein Assay Kit (Thermo Fisher Scientific, Waltham, MA). The protein mixture was resuspended in 450 μL of rehydration solution containing 7 M urea, 2 M thiourea, 2% CHAPS, 18 mM DTT, 2% pharmalyte pH 3–10 nonlinear (NL), and a trace of bromophenol blue. In total, 300 μg of proteins were loaded onto Immobiline DryStrip gel (IPG strips) (24 cm; pH 3–10 NL; GE Healthcare, Sweden) with passive rehydration for 12 h. Isoelectric focusing (IEF) was performed at 20 °C using an Ettan IPGphor apparatus (GE Healthcare, Uppsala, Sweden) with the current limited to 50 μA/strip and the following voltage program: Step 1 at 500 V for 2 h, step 2 at 1000 V for 1 h in gradient, step 3 at 10,000 V for 3 h in gradient, and step 4 at 10,000 V for 4 h. Prior to SDS-PAGE, the focused IPG strips were equilibrated first in 6 M urea, 75 mM Tris-HCl (pH 8.8), 2% sodium dodecyl sulfate, 29.3% glycerol, 0.002% bromophenol blue containing 65 mM DDT for 15 min, and then for 15 min in the same buffer, replacing DTT with 2.5% iodoacetamide. After equilibration, the strips were transferred to 12.5% SDS polyacrylamide gels (DALT Gel; a precast polyacrylamide gel; GE Healthcare, Uppsala, Sweden). Each equilibrated strip was sealed with warm 0.5% (w/v) agarose. For the assessment of biological variations, three biological replicates of each sample were examined. The gels were run at 1 W per gel for the first 1 h and then at 17 W per gel for approximately 4.5 h until the bromophenol blue front reached the bottom of each gel.

### 3.3. Image Analysis

After the 2-DE electrophoresis, gels were stained with Coomassie Brilliant Blue G-250, then were documented with an Image Scanner III (GE Healthcare, Uppsala, Sweden), and were saved as TIF images for further analysis. Spot detection, spot matching, and protein quantification were performed using SameSpots software (Totallab, Newcastle, UK). Spots whose abundance were determined to be altered by at least two-fold and exhibited a *p*-value < 0.05 from all replicates were considered as differentially regulated and were designated to mass spectrometry for identification. To show differences between the analyzed samples, principal component analysis was calculated by SameSpots software (Totallab, Newcastle, UK).

### 3.4. Spots Identification Using Matrix-Assisted Laser Desorption/ Ionization Time-of-Flight Tandem Mass Spectrometry

Spots containing the proteins of interest (2-fold, *p*-value < 0.05) were excised manually from the gels and subjected overnight to in gel-trypsin digestion using 10 μL of 0.2 μg/μL modified sequencing grade trypsin (Promega, Madison, WI) solution in 50 mM NH_4_HCO_3_. After that the spots were first washed with 100% acetonitrile (ACN), then equilibrated with 50% ACN in 0.1% trifluoroacetic acid (TFA) and 0.1% TFA in water, and eluted with 2 μL of 50% ACN in 0.1% TFA using Zip-Tip C18 tips (Millipore, Billerica, USA). Samples were then mixed with matrix solution consisting of 5 mg of α-cyano-4-hydroxycinnamic acid (Bruker Daltonics, Billerica, USA) in 1 mL of 50% acetonitrile in 0.1% trifluoroacetic acid. The mixture was transferred onto the MALDI target plate MT 34 Target Plate Ground Steel (Bruker Daltonics, Billerica, USA) and left to dry. Peptide mass spectra were acquired using a MALDI-TOF tandem mass spectrometer (Autoflex Speed, Bruker Daltonics). Up to 6 of the most intense ions were selected for MS/MS spectra acquisition. The collected spectra were analyzed using BioTools (Bruker Daltonics, Billerica, USA) and searched using BioTools as a front end. Then, MS peptide mass fingerprint (PMF) and fragment mass spectra (MS/MS) from each individual spot were combined and identified against the National Centre for Biotechnology Information database (NCBI) using an in-house Mascot Server (Matrix Science, London, UK). The *Pseudomonas putida* KT2440 database was searched using the MALDI TOF/TOF-MS data by specifying trypsin as the proteolytic enzyme, allowing two missed cleavages with carbamidomethyl cysteine as a fixed modification and oxidized methionine as a variable modification. Additionally, for the PMF and MS/MS ion search, the results were filtered with a statistically significant threshold of *p* < 0.05. At least two correctly identified parent ions by MASCOT with a MASCOT ion score cut-off of ≥ 30 were regarded as correct hits.

### 3.5. Statistical Analysis

The presented data are the mean with standard deviation (SD) of three different replicate experiments. Statistical analysis of the changes in protein abundance was performed using the SameSpots software. The protein spot maps corresponding to biological replicates were analyzed using one-way ANOVA. Changes in protein spot abundance were considered statistically significant at *p*-value < 0.05 and with at least a 2.0-fold change. Normalized spot intensities on gels were compared a Student’s t-test at a significance level of 0.05.

## 4. Conclusions

This study demonstrated the cellular responses of *Pseudomonas putida* KT2440 to dual carbon-phosphorus limitation at the proteomic level. In the obtained proteome profiles, many changes during the different growth phases and mcl-PHAs synthesis were observed. The results showed that mcl-PHAs were accumulated when carbon and phosphorus was in a limiting concentration. Severe metabolic and cellular reorganization was observed, suggesting that the cells’ response to the applied environmental stress has a multifaceted nature. The majority of proteins involved in carbon and energy metabolism were downregulated in the late stationary phase when mcl-PHAs were not synthesized, suggesting that most of the metabolic activities were ceased in the bacterial cells under carbon- and phosphorus-limiting conditions. However, the abundance of proteins related to amino acid catabolism seemed to be essential to generate energy. Furthermore, at the same time, upregulation of PhaF protein was observed, as a negative regulator of the *pha* cluster could repress the expression of other PHA-related proteins. To maintain an off-equilibrium condition, *P. putida* KT2440 induced some proteins involved in the transport of molecules across cellular membranes. It was also verified that nutrient limitation triggered the production of mcl-PHAs and influenced the expression of proteins related to DNA replication, transcription, and ribosome synthesis. Moreover, the experimental results indicate that dual carbon-phosphorus limitation and mcl-PHAs accumulated in the bacterial cells changed the protein abundance involved in stress response and signal transduction. The presented data allowed investigation of time-course proteome analysis in response to carbon and phosphorus limitation, and mcl-PHAs synthesis. The presented data expands the knowledge of the response of *Pseudomonas putida* KT2440 to limited carbon and phosphorus levels, opening up the potential for the design of more efficient mcl-PHAs synthesis processes.

## Supplementary Materials

The following are available online at https://www.mdpi.com/2218-273X/9/12/796/s1, Table S1: Parameters measured during the cultivation of *Pseudomonas putida* KT2440 under non-limiting conditions. The mean was taken from three biologically independent replicates. Table S2: Identification of differentially expressed proteins at 24 h and 48 h of the *Pseudomonas putida* KT2440 fermentation during mcl-PHAs synthesis.
