# Supplementary material for: Proteomic Response of Pseudomonas putida KT2440 to Dual Carbon-Phosphorus Limitation during mcl-PHAs Synthesis
Source: Biomolecules. 2019 Nov 28;9(12):796. doi: 10.3390/biom9120796 (PMC6995625; doi:10.3390/biom9120796)
Supplement: Supplementary file 1 [file biomolecules-09-00796-s001.zip › biomolecules-632155-supplementary/Table S1-supplementary material.docx]

Table S1. Parameters measured during the cultivation of *Pseudomonas putida* KT2440 under non-limiting conditions. The mean was taken from three biologically independent replicates.

| **Time** | **Biomass (g/L)** | **PHA (g/L)** | **Phosphorus concentration (mg/L)** | **Ammonium concentration (g/L)** |
| --- | --- | --- | --- | --- |
| 0 | 0.17 ± 0.01 | 0 | 462 ± 3.54 | 2.185 ± 0.02 |
| 8 | 2.39 ± 0.02 | 0 | 390 ± 3.05 | 1.915 ± 0.01 |
| 17 | 3.19 ± 0.01 | 0 | 260 ± 12.02 | 1.835 ± 0.01 |
| 24 | 3.06 ± 0.03 | 0 | 244 ± 6.36 | 1.605 ± 0.01 |
| 32 | 2.94 ± 0.02 | 0 | 236 ± 3.54 | 1.535 ± 0.03 |
| 41 | 2.80 ± 0.01 | 0 | 248 ± 8.49 | 1.520 ± 0.03 |
| 48 | 2.99 ± 0.04 | 0 | 246 ± 14.14 | 1.505 ± 0.05 |
