# Supplementary material for: Proteomic Response of Pseudomonas putida KT2440 to Dual Carbon-Phosphorus Limitation during mcl-PHAs Synthesis
Source: Biomolecules. 2019 Nov 28;9(12):796. doi: 10.3390/biom9120796 (PMC6995625; doi:10.3390/biom9120796)
Supplement: Supplementary file 1 [file biomolecules-09-00796-s001.zip › biomolecules-632155-supplementary/Table S2-supplementary material.docx]

| Spot no.  Table S2. Identification of differentially expressed proteins at 24 h and 48 h of the *Pseudomonas putida* KT2440 fermentation during mcl-PHAs synthesis | Identified protein | Accession number | Fold change 8 h vs 24 h | Fold change 8 h vs 48 h | Fold change 24 h vs 48 h | pI | MW (Da) | Protein score | Sequence coverage (%) |
| --- | --- | --- | --- | --- | --- | --- | --- | --- | --- |
| **Carbon metabolism** | | | | | | | | | |
| 4574 | acetyl-CoA carboxylase biotin carboxyl carrier protein subunit | gi\|26987297 | -3.9 | -4.0 | nd | 4.95 | 16232 | 197 | 28 |
| 1677 | phosphopyruvate hydratase | gi\|26988344 | -2.1 | nd | nd | 4.92 | 45633 | 77 | 8 |
| 1697 | enolase | gi\|26988344 | 2.4 | 2.5 | nd | 5.70 | 48065 | 127 | 9 |
| 3552 | carbohydrate kinase, thermoresistant glucokinase family | gi\|26990129 | -2.3 | -3.3 | nd | 5.58 | 19228 | 87 | 20 |
| 694 | isocitrate dehydrogenase, NADP-dependent | gi\|26990717 | -2.4 | -3.4 | nd | 5.44 | 80078 | 156 | 15 |
| 1271 | dihydrolipoamide dehydrogenase | gi\|26990879 | 2.1 | 2.5 | nd | 5.93 | 50109 | 216 | 22 |
| 1494 | branched-chain alpha-keto acid dehydrogenase complex dihydrolipoyl dehydrogenase | gi\|26991093 | 3.7 | 4.1 | nd | 6.25 | 48683 | 170 | 18 |
| 4571 | PfkB domain protein | gi\|26990093 | -3.4 | -4.0 | nd | 5.55 | 33879 | 149 | 53 |
| 2828 | alpha-ketoglutarate semialdehyde dehydrogenase | gi\|26987991 | 2.5 | 2.2 | nd | 5.93 | 57241 | 56 | 9 |
| 1544 | branched-chain alpha-keto acid dehydrogenase subunit E2 | gi\|26991092 | 2.6 | 2.6 | nd | 5.93 | 45249 | 213 | 22 |
| 1642 | UDP-N-acetylglucosamine pyrophosphorylase | gi\|26992086 | -6.5 | nd | nd | 6.24 | 48555 | 353 | 33 |
| 689 | hydroperoxidase II | gi\|26986860 | nd | 2.2 | nd | 6.21 | 78114 | 97 | 20 |
| 2261 | glyceraldehyde-3-phosphate dehydrogenase, type I | gi\|26987745 | nd | -2.1 | nd | 6.49 | 36320 | 273 | 50 |
| 1045 | phosphogluconate dehydratase | gi\|26987746 | nd | -2.1 | nd | 6.00 | 65774 | 311 | 37 |
| 502 | phosphoenolpyruvate synthase | gi\|26988807 | nd | -2.1 | nd | 5.02 | 86475 | 380 | 30 |
| 1727 | type II citrate synthase | gi\|26990886 | nd | -2.1 | nd | 6.50 | 47933 | 89 | 9 |
| 2153 | 2-ketogluconate 6-phosphate reductase | gi\|26990091 | nd | -2.2 | nd | 5.92 | 34768 | 155 | 52 |
| 1085 | gluconate 2-dehydrogenase acceptor subunit | gi\|26990098 | nd | -4.2 | nd | 7.68 | 65350 | 121 | 32 |
| 2333 | NAD-dependent epimerase/dehydratase | gi\|26987240 | nd | -3.0 | nd | 5.28 | 33532 | 151 | 37 |
| 883 | acetyl-CoA synthetase | gi\|26991173 | nd | -2.1 | nd | 5.94 | 72208 | 201 | 14 |
| **Energy metabolism** | | | | | | | | | |
| 1457 | F0F1 ATP synthase subunit beta | gi\|26992088 | nd | -3.2 | nd | 4.88 | 49385 | 314 | 35 |
| 1737 | glutamate dehydrogenase | gi\|26987411 | -3.4 | -4.2 | nd | 6.12 | 49296 | 144 | 51 |
| 2533 | nitrite reductase large subunit | gi\|26988437 | -2.6 | -2.6 | nd | 5.78 | 94095 | 84 | 11 |
| 1174 | glutamine synthetase, type I | gi\|26991722 | -3.0 | -2.9 | nd | 5.21 | 51936 | 207 | 35 |
| 370 | NADH dehydrogenase subunit G | gi\|26990818 | nd | -2.9 | nd | 5.69 | 98547 | 266 | 27 |
| 2382 | NAD synthetase | gi\|26991549 | 2.5 | 6.3 | 2.5 | 5.52 | 29426 | 378 | 31 |
| 3053 | nitroreductase | gi\|26991272 | -3.1 | nd | nd | 5.74 | 21983 | 219 | 54 |
| 290 | bifunctional aconitate hydratase 2/2-methylisocitrate dehydratase | gi\|26989063 | -2.1 | -2.4 | nd | 5.18 | 94178 | 210 | 17 |
| **PHA synthesis** | | | | | | | | | |
| 731 | polyhydroxyalkanoate granule-associated protein GA2 | gi\|26991684 | 2.8 | 2.4 | nd | 5.29 | 17928 | 60 | 51 |
| **Nucleotide metabolism** | | | | | | | | | |
| 1621 | ribonucleotide-diphosphate reductase subunit beta | gi\|26987912 | -2.8 | -3.0 | nd | 4.81 | 47301 | 323 | 52 |
| 259 | ribonucleotide-diphosphate reductase subunit alpha | gi\|26987914 | -3.7 | -3.5 | nd | 5.61 | 107452 | 193 | 20 |
| 1516 | bifunctional D-hydantoinase/dihydropyrimidinase | gi\|1002826252 | 2.3 | 3.1 | nd | 5.72 | 54790 | 316 | 24 |
| 1576 | dihydroorotase-like protein | gi\|26991676 | 2.3 | 2.1 | nd | 5.71 | 52766 | 139 | 39 |
| **Amino acids metabolism and biosynthesis** | | | | | | | | | |
| 2476 | 3-hydroxyisobutyrate dehydrogenase | gi\|26991350 | 2.6 | 3.2 | nd | 5.84 | 30530 | 154 | 41 |
| 1971 | leucine dehydrogenase | gi\|26991301 | 2.4 | 3.0 | nd | 6.25 | 36246 | 139 | 26 |
| 1899 | aminotransferase | gi\|26990491 | -4.5 | -3.7 | nd | 5.95 | 44610 | 262 | 57 |
| 2623 | diaminopimelate epimerase | gi\|26990495 | -2.1 | -2.3 | nd | 5.75 | 29781 | 183 | 60 |
| 2461 | N-acetyl-gamma-glutamyl-phosphate reductase | gi\|26990344 | -2.2 | -2.1 | nd | 6.22 | 33660 | 278 | 63 |
| 1040 | hydantoinase B/oxoprolinase | gi\|26990226 | -3.0 | -3.6 | nd | 5.46 | 63700 | 183 | 37 |
| 797 | 5-oxoprolinase (ATP-hydrolyzing) | gi\|26990227 | -3.8 | -4.1 | nd | 5.86 | 75115 | 241 | 50 |
| 2889 | phosphoribosyl-AMP cyclohydrolase | gi\|26991690 | 3.0 | 4.6 | nd | 6.05 | 15199 | 66 | 36 |
| 1071 | 2-isopropylmalate synthase | gi\|26987761 | nd | -2.3 | nd | 5.26 | 62016 | 73 | 21 |
| 2222 | cysteine synthase A | gi\|26991255 | nd | 2.2 | nd | 5.55 | 34507 | 64 | 16 |
| 459 | cysteine synthase B | gi\|148549270 | 2.1 | 2.2 | nd | 5.46 | 33870 | 30 | 28 |
| 2093 | 4-hydroxyphenylpyruvate dioxygenase | gi\|26990146 | nd | -3.2 | nd | 5.07 | 40075 | 287 | 23 |
| 2477 | homocysteine S-methyltransferase family protein | gi\|26987394 | -2.1 | -2.3 | nd | 5.15 | 33557 | 107 | 38 |
| 2804 | pyrroline-5-carboxylate reductase | gi\|26990483 | nd | -2.3 | nd | 5.84 | 27868 | 131 | 41 |
| **Lipopolysaccharide biosynthesis** | | | | | | | | | |
| 2041 | UDP-3-O-[3-hydroxymyristoyl] N-acetylglucosamine deacetylase | gi\|26988077 | 2.1 | 2.2 | nd | 5.06 | 33248 | 355 | 32 |
| 2576 | glucose-1-phosphate thymidylyltransferase | gi\|26988514 | nd | -2.1 | nd | 5.09 | 32668 | 183 | 63 |
| **Metabolism of cofactors and vitamins** | | | | | | | | | |
| 3176 | 3-methyl-2-oxobutanoate hydroxymethyltransferase | gi\|26991383 | 2.7 | 3.3 | nd | 5.47 | 27905 | 50 | 7 |
| 3319 | bacterioferritin | gi\|26987818 | 2.6 | 3.4 | nd | 4.74 | 18074 | 196 | 29 |
| **Metabolism and biosynthesis of terpenoids and polyketides** | | | | | | | | | |
| 3250 | dTDP-4-dehydrorhamnose 3,5-epimerase | gi\|26988513 | -5.2 | -6.5 | nd | 4.94 | 20323 | 165 | 46 |
| 2248 | hypothetical protein PP_3783 (syringomycin biosynthesis protein 2) | gi\|26990488 | -2.9 | nd | nd | 5.55 | 35030 | 274 | 66 |
| **Xenobiotics biodegradation and metabolism** | | | | | | | | | |
| 1830 | Oye family NADH-dependent flavin oxidoreductase | gi\|26989209 | -2.6 | -2.7 | nd | 6.04 | 41121 | 223 | 56 |
| **Transcription, translation, folding, sorting and degradation** | | | | | | | | | |
| 3797 | 50S ribosomal protein L9 | gi\|26991554 | nd | -2.2 | nd | 5.41 | 15457 | 279 | 63 |
| 505 | elongation factor Tu | gi\|26987193 | 3.6 | 2.6 | nd | 5.22 | 43793 | 65 | 26 |
| 1561 | elongation factor Tu-A | gi\|26987181 | 6.7 | nd | nd | 4.41 | 22034 | 65 | 8 |
| 2601 | transcriptional regulator Anr | gi\|26990956 | -2.4 | -2.4 | nd | 6.14 | 27489 | 219 | 25 |
| 1434 | C4-type zinc finger DksA/TraR family protein | gi\|26991377 | -2.1 | -2.0 | nd | 5.60 | 17349 | 74 | 20 |
| 825 | tRNA uridine 5-carboxymethylaminomethyl modification enzyme GidA | gi\|26986749 | -2.5 | -3.7 | nd | 6.03 | 69867 | 70 | 20 |
| 717 | polynucleotide phosphorylase/polyadenylase | gi\|26991392 | nd | -2.1 | nd | 5.12 | 75192 | 147 | 38 |
| 3280 | transcription elongation factor GreA | gi\|26991405 | nd | -2.5 | nd | 4.85 | 17564 | 190 | 66 |
| 2304 | LysR family transcriptional regulator | gi\|26990484 | -6.2 | -6.4 | nd | 6.41 | 34069 | 93 | 40 |
| 1751 | LysR family transcriptional regulator | gi\|26991976 | 2.9 | nd | nd | 7.71 | 33211 | 56 | 19 |
| 4572 | elongation factor 4 | gi\|26988164 | nd | 2.4 | 2.5 | 5.58 | 66602 | 83 | 6 |
| 771 | transcription elongation factor NusA | gi\|26991396 | nd | nd | -3.3 | 4.58 | 54697 | 61 | 13 |
| 3742 | YbaK/prolyl-tRNA synthetase associated region | gi\|26986945 | nd | 4.1 | 2.2 | 6.29 | 18483 | 276 | 45 |
| **Replication and repair** | | | | | | | | | |
| 3258 | single-strand DNA-binding protein | gi\|26987226 | nd | -2.1 | nd | 5.88 | 20161 | 88 | 22 |
| 2591 | DNA-3-methyladenine glycosylase | gi\|26987441 | nd | 2.5 | nd | 4.84 | 35271 | 50 | 11 |
| 369 | DNA gyrase subunit B | gi\|26986758 | nd | -2.6 | nd | 5.64 | 90203 | 94 | 7 |
| **Chaperones (lepiej stress response)** | | | | | | | | | |
| 1058 | chaperonin GroEL | gi\|26988095 | 5.4 | 5.2 | nd | 5.49 | 58098 | 167 | 12 |
| 804 | chaperone protein HscA | gi\|26987582 | -2.5 | -2.1 | nd | 4.92 | 66019 | 198 | 55 |
| 675 | chaperone protein DnaK | gi\|26991410 | nd | -2.3 | nd | 4.83 | 68873 | 105 | 37 |
| 3793 | heat shock protein Hsp20 | gi\|26988708 | nd | -2.6 | nd | 5.94 | 16329 | 192 | 79 |
| 2963 | peptidyl-prolyl cis-trans isomerase, FKBP-type | gi\|26987420 | nd | -2.3 | nd | 4.64 | 21665 | 129 | 54 |
| 3811 | universal stress protein | gi\|26988856 | 2.7 | 3.5 | nd | 6.14 | 16369 | 140 | 32 |
| 3541 | DNA-binding stress protein, putative | gi\|26987945 | 2.1 | nd | nd | 5.20 | 17850 | 417 | 59 |
| 1372 | PhoH family protein | gi\|26988026 | nd | 2.1 | nd | 5.54 | 52052 | 337 | 21 |
| 3051 | anti-oxidant AhpCTSA family protein | gi\|26987820 | nd | -2.1 | nd | 5.06 | 21887 | 348 | 81 |
| 1001 | quinoprotein ethanol dehydrogenase | gi\|26989393 | nd | 2.3 | nd | 6.52 | 69244 | 96 | 17 |
| **Transport** | | | | | | | | | |
| 1926 | ABC transporter ATP-binding protein | gi\|26987814 | -2.1 | -2.3 | nd | 5.96 | 40390 | 131 | 39 |
| 2726 | OmpF family protein | gi\|26988814 | nd | 7.2 | 3.9 | 4.72 | 37217 | 122 | 20 |
| 2836 | OmpF family protein | gi\|26988814 | 2.6 | 4.4 | nd | 4.72 | 37217 | 135 | 14 |
| 2413 | OmpF family protein | gi\|26988814 | 3.3 | nd | nd | 4.72 | 37217 | 94 | 14 |
| 2319 | OmpF family protein | gi\|26988814 | 2.2 | nd | -2.3 | 4.72 | 37217 | 78 | 25 |
| 2871 | OmpF family protein | gi\|26988814 | nd | 2.3 | nd | 4.72 | 37217 | 108 | 14 |
| 3355 | polysaccharide export protein | gi\|26989845 | 3.9 | 3.4 | nd | 5.15 | 19572 | 119 | 16 |
| 1838 | extracellular ligand-binding receptor | gi\|26987877 | 2.2 | nd | nd | 6.02 | 39491 | 191 | 27 |
| 1840 | BraC-like branched-chain amino acid ABC transporter substrate-binding protein | gi\|26991547 | 2.5 | nd | nd | 5.93 | 40417 | 88 | 33 |
| 2982 | sulfur compound ABC transporter ATP-binding protein | gi\|26986968 | nd | 2.4 | nd | 8.94 | 28071 | 52 | 24 |
| 1275 | dipeptide ABC transporter, periplasmic peptide-binding protein | gi\|26987621 | nd | 2.1 | nd | 6.64 | 60556 | 68 | 15 |
| 2287 | general amino acid ABC transporter, periplasmic binding protein | gi\|26988032 | nd | 2.1 | nd | 5.84 | 36630 | 183 | 16 |
| 2174 | iron ABC transporter, periplasmic iron-binding protein, putative | gi\|26991560 | nd | -2.2 | nd | 5.27 | 36777 | 277 | 48 |
| 2127 | ABC transporter, periplasmic polyamine-binding protein, putative | gi\|26992017 | nd | 2.1 | nd | 8.59 | 40756 | 54 | 15 |
| 1895 | translocation protein TolB | gi\|26987957 | nd | 3.8 | nd | 9.44 | 47515 | 206 | 51 |
| 2602 | amino acid ABC transporter, periplasmic amino acid-binding protein | gi\|26990304 | 2.1 | nd | nd | 4.95 | 27527 | 78 | 17 |
| **Signal transduction** | | | | | | | | | |
| 2578 | LytTR family two component transcriptional regulator | gi\|26986929 | nd | 2.2 | nd | 6.09 | 27520 | 117 | 11 |
| 575 | sensor histidine kinase | gi\|26990086 | 4.4 | 4.8 | nd | 6.24 | 49499 | 79 | 6 |
| 2966 | LuxR family two component transcriptional regulator | gi\|26988367 | nd | 2.4 | nd | 5.06 | 23523 | 111 | 31 |
| **Cell motility (albo lepiej cellular processes)** | | | | | | | | | |
| 941 | flagellin FliC | gi\|26991067 | -4.9 | -12.5 | -2.6 | 4.39 | 67806 | 334 | 13 |
| 1051 | flagellin FliC | gi\|26991067 | nd | -2.4 | -2.7 | 4.39 | 67806 | 109 | 6 |
| 1537 | flagellar cap protein FliD | gi\|26991065 | -4.4 | -6.0 | nd | 5.50 | 46494 | 79 | 18 |
| 4573 | flagellar hook-associated protein FlgL | gi\|26991069 | nd | -4.2 | nd | 4.49 | 54905 | 345 | 40 |
| 1834 | cell division protein FtsZ | gi\|26988076 | nd | -2.1 | nd | 4.98 | 41918 | 177 | 43 |
| **Peptidoglycan biosynthesis and degradation** | | | | | | | | | |
| 2224 | ErfK/YbiS/YcfS/YnhG family protein | gi\|26989044 | -2.7 | -4.1 | nd | 5.44 | 35086 | 54 | 19 |
| **Other functions** | | | | | | | | | |
| 2698 | oxidoreductase FAD/NAD(P)-binding domain protein | gi\|26988370 | -2.1 | -2.1 | nd | 5.22 | 29805 | 207 | 56 |
| 2953 | hypothetical protein PP_5426 | gi\|1002825823 | -2.4 | -4.4 | nd | 4.90 | 23914 | 278 | 44 |
| 1631 | oxygen-independent coproporphyrinogen III oxidase family protein | gi\|26990486 | -8.1 | -7.3 | nd | 6.53 | 53323 | 159 | 39 |
| 2617 | hypothetical protein PP_3787 | gi\|26990492 | -2.6 | -2.8 | nd | 6.35 | 33650 | 189 | 50 |
| 2851 | dienelactone hydrolase | gi\|26987919 | 2.4 | 2.5 | nd | 6.30 | 28818 | 161 | 40 |
| 2459 | hypothetical protein PP_1795 | gi\|26988526 | -4.5 | -3.9 | nd | 5.97 | 31658 | 164 | 20 |
| 3204 | isochorismatase superfamily hydrolase | gi\|26988957 | 2.1 | 4.0 | 2.1 | 5.58 | 19398 | 232 | 43 |
| 2940 | HAD superfamily hydrolase | gi\|26988520 | -3.9 | -3.4 | nd | 5.01 | 25469 | 100 | 43 |
| 590 | hypothetical protein PP_1246 | gi\|26987981 | -3.1 | -5.5 | nd | 5.84 | 66310 | 195 | 9 |
| 4049 | hypothetical protein PP_0998 | gi\|26987734 | 3.3 | 4.2 | nd | 6.13 | 16083 | 103 | 49 |
| 1882 | PAS/PAC sensor hybrid histidine kinase | gi\|26989383 | 2.9 | 2.7 | nd | 6.19 | 73505 | 45 | 22 |
| 1884 | hypothetical protein PP_3784 | gi\|26990489 | -2.4 | -3.0 | nd | 5.43 | 41444 | 208 | 41 |
| 3237 | hypothetical protein PP_3611 | gi\|26990322 | 2.4 | 2.3 | nd | 9.07 | 20811 | 321 | 48 |
| 3589 | hypothetical protein PP_0797 | gi\|26987533 | 3.2 | 5.6 | nd | 6.32 | 20084 | 96 | 35 |
| 2682 | protein-tyrosine kinase | gi\|26989847 | 2.9 | 2.9 | nd | 6.60 | 29833 | 272 | 38 |
| 2768 | xylose isomerase domain protein TIM barrel | gi\|26990094 | -2.6 | -2.3 | nd | 5.40 | 28249 | 211 | 39 |
| 1575 | hypothetical protein PP_3785 | gi\|26990490 | -4.2 | -4.7 | nd | 6.06 | 51034 | 193 | 67 |
| 2400 | hypothetical protein PP_3777 | gi\|26990482 | -2.4 | -2.5 | nd | 5.31 | 31476 | 117 | 52 |
| 1721 | cobalamin biosynthesis protein CobW | gi\|26990220 | -2.2 | -2.6 | nd | 4.82 | 38898 | 104 | 26 |
| 2915 | ThiJ/PfpI domain protein | gi\|26990144 | 2.1 | nd | nd | 6.08 | 25344 | 239 | 29 |
| 924 | oligopeptidase A | gi\|26986841 | -2.1 | -2.9 | nd | 5.20 | 76016 | 246 | 44 |
| 3798 | CBS domain-containing protein | gi\|26986946 | 2.4 | 2.2 | nd | 6.84 | 16095 | 137 | 66 |
| 2589 | hypothetical protein PP_0694 | gi\|26987430 | -2.4 | -2.4 | nd | 4.52 | 27033 | 195 | 51 |
| 3750 | hypothetical protein PP_3241 | gi\|26989959 | nd | 3.3 | nd | 5.75 | 18249 | 182 | 21 |
| 2621 | PhzF family phenazine biosynthesis protein | gi\|26991006 | nd | 4.6 | 3.6 | 4.79 | 28591 | 142 | 17 |
| 3758 | hypothetical protein PP_0536 | gi\|26987274 | nd | 2.8 | nd | 5.66 | 18316 | 95 | 53 |
| 3038 | NAD-dependent epimerase/dehydratase | gi\|26988126 | nd | 2.2 | nd | 7.08 | 21972 | 102 | 17 |
| 2285 | carboxyvinyl-carboxyphosphonate phosphorylmutase, putative | gi\|26988242 | nd | 2.6 | nd | 4.48 | 29226 | 118 | 50 |
| 3501 | Sel1 domain protein repeat-containing protein | gi\|26991465 | nd | 2.4 | nd | 4.82 | 19889 | 73 | 20 |
| 1938 | hypothetical protein PP_1631 | gi\|26988363 | nd | 2.1 | nd | 6.18 | 42231 | 215 | 40 |

nd – not detected as significantly differentially expressed
